# EEG potentials associated with artificial grammar learning in the primate brain

**DOI:** 10.1016/j.bandl.2014.11.006

**Published:** 2015-09

**Authors:** Adam Attaheri, Yukiko Kikuchi, Alice E. Milne, Benjamin Wilson, Kai Alter, Christopher I. Petkov

**Affiliations:** a Institute of Neuroscience, Henry Wellcome Building, Newcastle University, Framlington Place, Newcastle upon Tyne NE2 4HH, United Kingdom; b Centre for Behaviour and Evolution, Henry Wellcome Building, Newcastle University, Framlington Place, Newcastle upon Tyne NE2 4HH, United Kingdom

**Keywords:** Monkey, mMMN, Primate, Comparative neurobiology, Electroencephalography (EEG), Event Related Potentials (ERPs), Communication, Language, Statistical learning

## Abstract

Electroencephalography (EEG) has identified human brain potentials elicited by Artificial Grammar (AG) learning paradigms, which present participants with rule-based sequences of stimuli. Nonhuman animals are sensitive to certain AGs; therefore, evaluating which EEG Event Related Potentials (ERPs) are associated with AG learning in nonhuman animals could identify evolutionarily conserved processes. We recorded EEG potentials during an auditory AG learning experiment in two Rhesus macaques. The animals were first exposed to sequences of nonsense words generated by the AG. Then surface-based ERPs were recorded in response to sequences that were ‘consistent’ with the AG and ‘violation’ sequences containing illegal transitions. The AG violations strongly modulated an early component, potentially homologous to the Mismatch Negativity (mMMN), a P200 and a late frontal positivity (P500). The macaque P500 is similar in polarity and time of occurrence to a late EEG positivity reported in human AG learning studies but might differ in functional role.

## Highlights

• First combined EEG and Artificial Grammar (AG) learning study in nonhuman animals.
• Early and late frontal potentials modulated in response to violations of the AG sequencing relationships in the primate brain.
• Informative similarities and differences are noted in relation to reported human EEG potentials associated with AG learning.

## 1 Introduction

To better understand the neurobiology of human language requires distinguishing language-specific processes from cognitive, domain-general processes not restricted to language (Bickerton & Szathmary, 2009; Fedorenko, Duncan, & Kanwisher, 2012; Friederici, 2011; Hagoort, 2005). Certain domain-general processes may be evolutionarily conserved (Bozic, Tyler, Ives, Randall, & Marslen-Wilson, 2010; Fitch & Friederici, 2012). Thus insights into how the human brain has specialised for language could come from cross-species comparative neurobiology. However, all comparative efforts depend on first finding evidence for shared abilities on tasks thought to be associated with language-related processes in humans and second, testing nonhuman animals using neurobiological techniques commonly used in humans.

Artificial Grammars (AG) regulate the relationships between stimuli in a sequence (Friederici, Bahlmann, Heim, Schubotz, & Anwander, 2006; Gomez & Gerken, 1999; Marcus, Vijayan, Bandi Rao, & Vishton, 1999; Reber, 1967; Saffran, 2002; Saffran, Johnson, Aslin, & Newport, 1999). AG learning paradigms have been used to explore the abilities of adult humans, pre-verbal infants and nonhuman animals to process different AGs (Bahlmann, Schubotz, & Friederici, 2008; Fitch & Hauser, 2004; Gentner, Fenn, Margoliash, & Nusbaum, 2006; Murphy, Mondragon, & Murphy, 2008; Saffran et al., 2008; van Heijningen, de Visser, Zuidema, & ten Cate, 2009; Wilson et al., 2013). Typically, in AG learning studies the participant is exposed to exemplary sequences of stimuli generated by the AG. In a subsequent testing phase, the participant’s responses to ‘consistent’ sequences that follow the AG are evaluated relative to those that violate it. Different responses to ‘violation’ versus consistent sequences can provide evidence that the participant is sensitive to the properties of the AG.

The behavioural literature has highlighted potentially conserved capabilities associated with AG learning in humans and other animals. Nonhuman animals, including primates, have been shown to be sensitive to a range of simple and moderate complexity AGs (Fitch & Hauser, 2004; Gentner et al., 2006; Hauser & Glynn, 2009; Murphy et al., 2008; Saffran et al., 2008; van Heijningen et al., 2009; Wilson et al., 2013). In this regard, even relatively simple AGs (such as ‘finite-state’ AGs, which generate a finite set of sequences based on adjacent transitions between stimuli; Chomsky, 1957; Fitch & Friederici, 2012) have been informative about evolutionarily conserved sequence processing capacities that predated human language evolution. Concurrently, human brain imaging studies with functional Magnetic Resonance Imaging (fMRI) have demonstrated that much of the perisylvian language network can also be engaged during AG sequence processing (Bahlmann et al., 2008; Friederici, 2011; Friederici et al., 2006; Petersson, Folia, & Hagoort, 2012).

Although fMRI can identify brain regions associated with AG learning, the temporal resolution of EEG is better suited for identifying the time course of the neural response to AG sequences. In humans, the polarity and general topography of EEG Event Related Potentials (ERPs) associated with AG learning have been identified and related to neurophysiological markers of natural language-related processes (Friederici, 2002, 2005). Moreover, EEG studies in humans and other animals have identified ERP components associated with ‘oddball’ or change detection paradigms, which can also be modulated by AG learning paradigms. Specifically, unexpected auditory oddball stimuli elicit a Mismatch Negativity (MMN; or its monkey homolog: mMMN), which is an enhanced negativity at ∼150 ms thought to be generated by regions including auditory cortex (Bekinschtein et al., 2009; Fishman & Steinschneider, 2012; Gil-da-Costa, Stoner, Fung, & Albright, 2013; Javitt, Schroeder, Steinschneider, Arezzo, & Vaughan, 1992; Naatanen & Alho, 1995; Ueno et al., 2008; Uhrig, Dehaene, & Jarraya, 2014; Ulanovsky, Las, & Nelken, 2003). The human P200 is an ERP component that can be modulated by attention to infrequently presented auditory targets (Garcia-Larrea, Lukaszewicz, & Mauguiere, 1992; Novak, Ritter, & Vaughan, 1992). A positivity at ∼300 ms (P3a) elicited in humans and its homolog in nonhuman primates is thought to be a more general marker of change detection that seems to involve dorsal fronto-central brain regions (Arthur & Starr, 1984; Gil-da-Costa et al., 2013; Molholm, Martinez, Ritter, Javitt, & Foxe, 2005; Paller, Zola-Morgan, Squire, & Hillyard, 1988; Pineda, Foote, Neville, & Holmes, 1988; Zevin, Yang, Skipper, & McCandliss, 2010). The MMN and P3a components in humans can also be elicited by AG paradigms, especially if violation sequences are presented infrequently (Baldwin & Kutas, 1997; Mueller, Friederici, & Männel, 2012). Moreover, in humans AG learning paradigms can influence other ERP components also associated with natural language processes (Friederici, 2004; Friederici, Steinhauer, & Pfeifer, 2002). Namely, violations to adjacent AG relationships (local violations) elicit more negative potentials at ∼200–350 ms, such as the Early Left Anterior Negativity (ELAN), which is stronger in frontal electrodes of the left hemisphere (Friederici, 2005). More complex AGs (such as those having non-adjacent relationships) can also elicit late centro-parietal positivities (P600: Friederici, 2004; Friederici et al., 2002).

Despite behavioural evidence in humans and other primates for finite-state AG learning, EEG studies associated with AG learning were not previously available in nonhuman primates. Therefore, the question posed is which macaque ERP components would be elicited by AG sequences? If certain neural processes for AG learning were evolutionarily conserved, we might expect to find macaque ERP responses similar to those reported for comparable AG processing in humans, including homologs of the MMN and ELAN potentials, some of which might be stronger in frontal electrodes.

## 2 Methods

### 2.1 Properties of the current AG and summary of prior macaque behavioural results

**Fig. 1:**
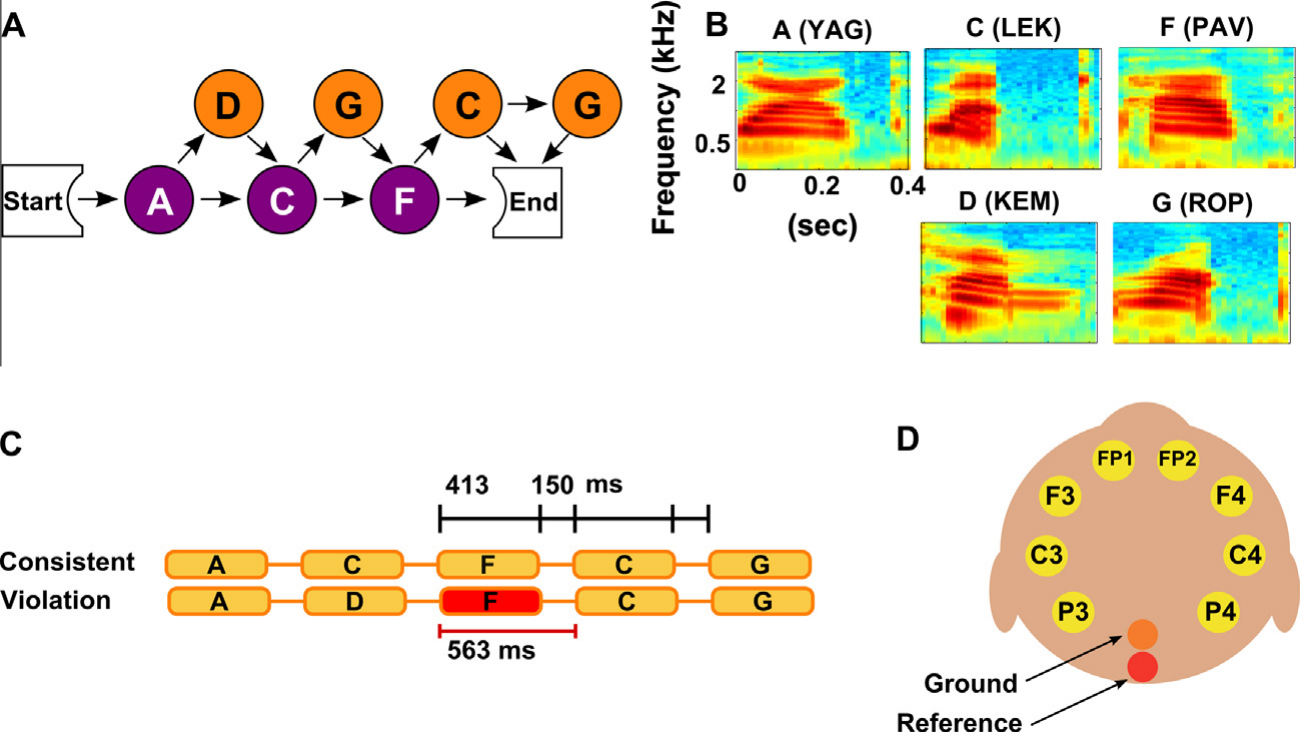
Artificial Grammar (AG), stimuli, sequence comparisons and macaque EEG electrode placement. (A) Schematic of the AG used. Following arrows from ‘start’ to ‘end’ creates a legal, consistent sequence. Not following the arrows (e.g., a ‘D’ to ‘F’ transition) creates a violation. (B) Spectrograms of the acoustic nonsense word sound elements (A, C, D, F, G) in the sequences. For example, the nonsense word “YAG” took the position of element A in the AG sequences. (C) Exemplary matching consistent vs. violation comparison sequence pair (see Suppl. Fig. S3 for all comparison pairs). The red box highlights the first illegal sound element in the violation sequence. The sequences are aligned so that acoustically identical elements can be compared (e.g., ‘F’, ‘C’ and ‘G’). (D) Illustrates the approximate location of the eight scalp surface EEG electrodes on the macaque, including ground and reference electrodes.

In a previous behavioural study we showed that Rhesus macaques are sensitive to a moderately complex finite-state AG (Wilson et al., 2013), based on an AG developed by Saffran et al. (2008). Such findings suggest that the cognitive abilities required for this form of AG learning are neither unique to humans nor to language. The AG consists of branching relationships between several obligatory and optional elements, all of which contribute to the structure of the AG (Fig. 1A). Such AGs allow the generation of less predictable (non-deterministic) sequences of varying length: a property of both sequences of natural events and of most linguistic sequences (Hurford, 2012; Petkov & Wilson, 2012). After a period of exposure to exemplary AG sequences, Rhesus macaques (two of which participated in the current EEG experiment) showed stronger orienting responses to novel sequences that violated the AG, relative to those that were ‘consistent’ with the AG (Wilson et al., 2013). In the behavioural work we also show evidence that the macaque AG learning results cannot be explained by simple learning strategies (Wilson et al., 2013).

### 2.2 Participants

Two adult male Rhesus monkeys (*Macaca mulatta*) from a group housed colony participated in this experiment (ages, Macaque 1 (M1) = 15 years, Macaque 2 (M2) = 7 years; weight: M1 = 9.8 kg, M2 = 16 kg). All the procedures performed were approved by the UK Home Office and comply with the Animal Scientific Procedures Act (1986) on the care and use of animals in research and also with the European Directive on the protection of animals used in research (2010/60/EU). We support the Animal Research Reporting of In Vivo Experiments (ARRIVE) principles on reporting animal research. All persons involved in this project were Home Office certified and the work was strictly regulated by the U.K. Home Office.

### 2.3 Stimuli

Each of the stimulus sequences (see Fig. 1) were made by digitally combining recordings of naturally spoken nonsense words produced by a female speaker. The recordings were made with an Edirol R-09HR (Roland Corp.) sound recorder. The amplitude of the recorded sounds was root-mean-square (RMS) balanced and the nonsense word stimuli were combined into sequences using customised Matlab scripts (150 ms inter-stimulus intervals). The experiments were coded in Matlab (Psychophysics Toolbox: http://psychtoolbox.org/) and Cortex software (Salk Institute). All sounds were presented at ∼75 dB SPL (calibrated with an XL2 sound level meter, NTI Audio). The durations of the spoken nonsense word stimuli were subsampled from a corpus so that all the stimuli (YAG, LEK, KEM, PAV, ROP) were 413 ms long. For additional details on the behavioural paradigm see Wilson et al. (2013). The experimental paradigm is shown in Fig. 1 and Suppl. Figs. S1–S3.

### 2.4 EEG recordings

The macaques were individually tested in a custom made primate chair within an acoustically insulated room (IAC). Once in the acoustic chamber the macaque was placed ∼60 cm in front of a monitor (24″ Samsung, LCD) on which a fixation spot was presented and the animal completed trials of fixation (Suppl. Fig. S1). The sounds were presented free-field from two audio speakers (Creative Inspire T10) placed horizontally at ±30° on either side of the monitor. A head post was used for immobilising the head during testing. EEG signals were recorded using eight Ag/AgCl (Silver/Silver-Chloride) electrodes held in place by a custom made cap (Fig. 1C). Signals were sampled at a rate of 1000 Hz through an EEG head stage and amplifier (Neuroscan).

### 2.5 Time course of a recording session

The macaques had previously been slowly acclimated to periods of head immobilisation and were trained using operant training and positive reinforcement to conduct a visual fixation task during sound stimulation. During the first phase of the experiment the animals were exposed for 30 min with the exemplary consistent AG sequences (Suppl. Fig. S2A). The exposure phase was followed by a ∼30 min testing phase (240 completed test sequence trials) where randomly selected consistent and violation testing sequences were individually presented (Suppl. Figs. S1–2). A test sequence trial was initiated by the animal fixating a spot at the centre of the screen, measured with an infra-red eye tracker (Arrington Research). To minimise eye movements, fixation to the centre of the screen had to be maintained throughout the test sequence presentation for a trial to be correctly completed (Suppl. Fig. S1). After the end of the test sequence trial there was a 1000 ms interval before the fixation spot was removed and the juice reward was given for a completed trial. Only completed trials were included for analysis with each session having a minimum of 240 completed trials. M1 broke fixation on average in 7% of the trials, M2 broke fixation on average in 10% of the trials. After the inter-trial-interval (minimally 4500 ms; Suppl. Fig. S1) the fixation spot re-appeared and the next testing trial began when the macaque engaged the fixation spot. See Suppl. Figs. S1–3 for illustrations of the trial timing and further details on the testing sequences used.

We had more testing sequences than we could test in a given session, as each testing session was completed at the macaque’s own pace. So to avoid undersampling the EEG data collection for any of the pairs of sequences we split the experimental data collection into two blocks, Block A and Block B. Each block contained the testing sequences illustrated in Suppl. Fig. S2 and consisted of a number of separate daily testing sessions (total number of sessions 74, M1 = 37 sessions, 20 with Block A, 17 with Block B; M2 = 37 sessions, 18 with Block A, 19 with Block B; see Suppl. Fig. S2). We counterbalanced the order of these two blocks of data collection between the two macaques (e.g., one macaque was tested first on Block A and the other on Block B).

### 2.6 EEG data analysis

#### 2.6.1 Pre-processing

Data analysis was conducted in Matlab R2011a (The Mathworks) using the EEGLAB toolbox (http://sccn.ucsd.edu/). Pre-processing was applied to the data from every channel in each recording session. First a high pass filter at 0.3 Hz and a notch filter at 50 Hz were applied to remove line noise effects. Manual inspection identified other noisy periods in the EEG trace which were removed. This resulted in on average 117 trials (±37 trials, standard deviation) remaining for further analysis, out of the available 240 trials per session. Following this, an independent-components algorithm (‘runica’ in EEGLAB) was used to identify any other artefacts, which in turn were extracted from the data.

The EEG activity elicited by each sequence trial within a session was epoched from 200 ms before the sequence onset through to 3250 ms after sequence onset. For each session an average waveform was created per channel for each of the sixteen sequences (Block A = four consistent and four violation sequences, Block B = four consistent and four violation sequences). This procedure was repeated for each macaque. Then for each average sequence waveform the 200 ms pre-sequence silent period was used for baseline correction. This baseline correction was repeated separately for each of the sixteen sequences. Subsequently, Block A and Block B sequences were treated as a combined set of sixteen sequences (eight consistent and eight violation) in all further analyses, unless specified otherwise. Finally, the average waveforms across sessions in the channels of interest were averaged creating a sequence average waveform.

#### 2.6.2 Analysis of consistent vs. violation sequence effects

Next each of the EEG waveforms in response to the violation sequences were analysed relative to the EEG responses to the matching consistent sequence pairs. This allowed us to compare the effects in response to a violation element in the violation sequences to an acoustically identical element in the consistent sequence (Fig. 1C). For this analysis some sequences had to be aligned so that acoustically matched periods of the sequences could be compared (Suppl. Fig. S3). After this alignment of sequences, the EEG responses to the comparison (violation or consistent) sequences were separately averaged (Fig. 3A). Then the consistent and violation sequence response waveforms were subtracted from each other to create a difference plot (violation minus consistent; Fig. 3B). From this difference waveform we computed the lower and upper bounds of the 95% confidence interval of the baseline period. This was defined based on the variability in the 613 ms time period, including the 200 ms before the onset of the sound sequence, through to the offset of the first sound element in the sequence, which was always the same ‘A’ element (−200 to 413 ms: 613 ms window). Therefore, the confidence interval (CI) reflects the 95% upper and lower bounds of the variability in the difference waveform during the baseline period (i.e., reflecting the 2.5% and 97.5% levels of the null difference waveform distribution). The CI analysis was used to identify significant differences between the matched violation and consistent sequence pairs (Fig. 3B).

To investigate the topographical distribution of the results, such as whether the effects identified with the CI analyses are more left or right hemisphere lateralised, or more anterior or posterior, we submitted the effects to a Repeated Measures ANOVA. First we obtained the time of the peak difference for the mMMN, P200 and P500, where the ERP components of interest maximally breached the CIs (e.g., Fig. 3B and Suppl. Fig. S4B). A 40 ms analysis window was centred on these time points (analysis windows used: mMMN: 128–168 ms; P200: 161–201 ms; P500: 480–520 ms). These analysis windows were then used to calculate the maximum EEG response value within the window, session-by-session. Next, the EEG voltage potentials in response to the ‘consistent’ and ‘violation’ analyses were submitted to the RM-ANOVA as a within-subject (repeated-measures) factor of ‘condition’ (consistent or violation). The RM-ANOVA also included the between-subjects factor of ‘Macaque’ (M1 or M2), and two other between-subjects factors of ‘Hemisphere’ (left or right) and ‘Antero-posterior axis’ (anterior or posterior). The total number of sessions numbered 74, M1 = 37 sessions (20 with Block A, 17 with Block B); M2 = 37 sessions (18 with Block A, 19 with Block B; Suppl. Fig. S3). Each session had two comparison sequence pairs (Suppl. Fig. S3) and data from the 8 channels. The ANOVA was conducted separately for the mMMN, P200 and P500 ERP components.

The Supplementary Materials reports additional supporting analyses and results as follows: (1) grand average responses in all electrodes (Suppl. Fig. S4); (2) ERP effects to the violating sound and a lack of such effects to the subsequent sound in the sequence (Suppl. Fig. S5); (3) analyses on whether the effects remain in sequences including no shifting between consistent and violation pairs and those that were balanced in the direction of shifting (Suppl. Fig. S6); (4) analyses showing that violation-related effects do not seem to depend on the EEG responses to the sound prior to the violation (Suppl. Fig. S7); and (5) ERP results shown separately by macaque (Suppl. Fig. S8) shown also in Table S1 as maximum voltages across sessions for the two macaques separately.

## 3 Results

We first exposed the macaques to the exemplary AG sequences for 30 min (Suppl. Fig. S2). Then in the subsequent testing phase, we recorded surface-based EEG potentials in the animals (Fig. 1D; Suppl. Fig. S1) as the animals listened to randomly presented testing sequences (Suppl. Fig. S2). The macaques conducted a fixation task during sequence presentation and EEG data acquisition (Suppl. Fig. S1). The testing sequences were either ‘consistent’ sequences generated by the AG or ‘violation’ sequences, which violated the AG structure by having an illegal transition between nonsense word ‘elements’ (Fig. 1C shows one consistent and violation matching sequence pair; see Suppl. Figs. S2-3 for all sequences used). Critically, the first illegal sound element in a violation sequence was identical to one in the matching consistent sequence, allowing us to analyse acoustically identical parts of the consistent and violation sequences (Suppl. Fig. S3).

**Fig. 2:**
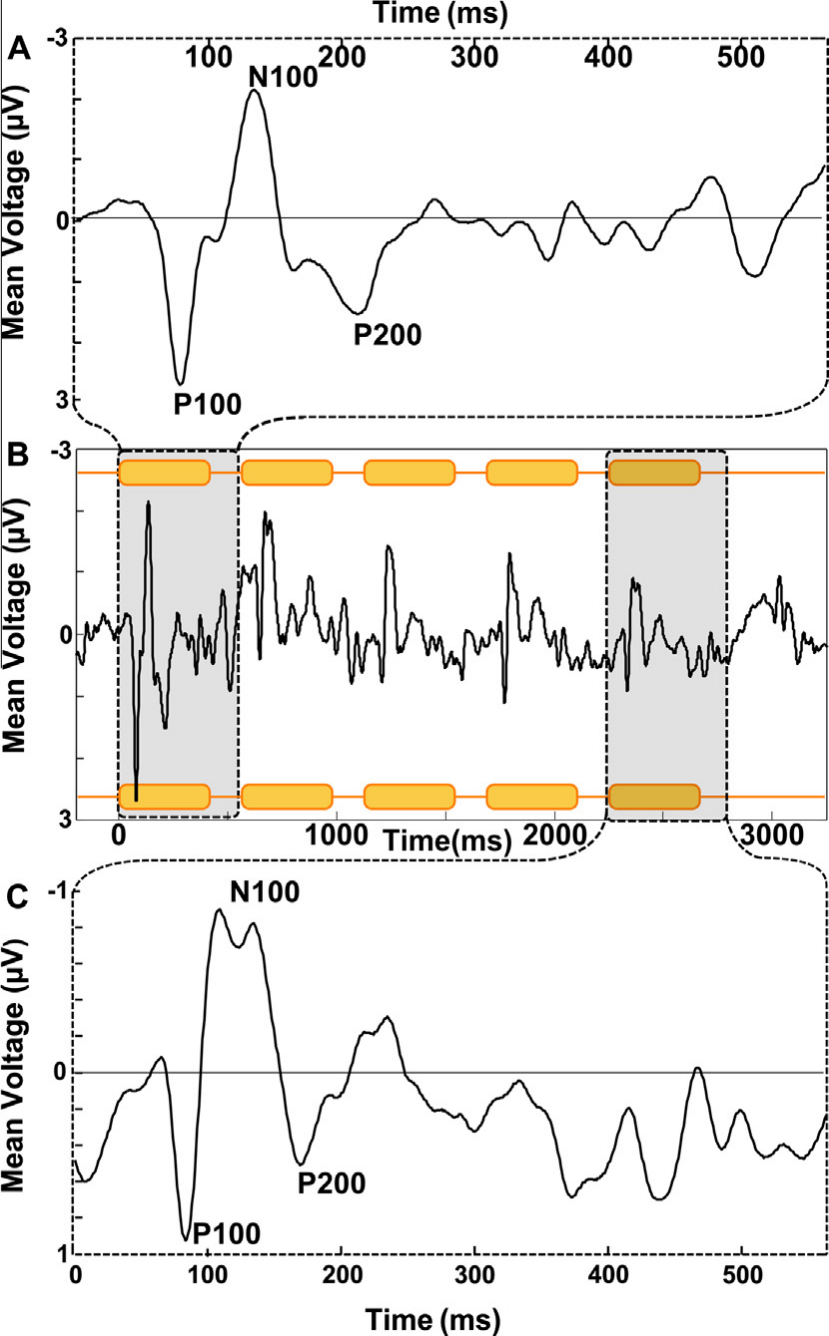
Event-Related Potentials (ERPs) in response to the sounds in the sequence. Grand average ERP from all electrodes (FP1, FP2, F3, F4, P3, P4, C3, C4) in response to all recorded sequences, all having five elements in a sequence. (A) Expanded ERP to the first element of the five within the sequences. (B) Grand average ERP to the five element long sequence. (C) Expanded ERP to the final element of the five in the sequence. Yellow boxes depict the periods of the ERP where the nonsense word elements were presented.

We first evaluated the EEG response to each of the 5 elements in the sequence and observed the presence of several typical Event Related Potential components (ERPs) to each of the elements in the sequence (Fig. 2). For this, we calculated the grand average ERP response to each of the elements (across all testing sequences, sessions, macaques and electrodes; Macaque 1, 37 testing sessions, Macaque 2, 37 testing sessions). The results show the presence of early positivities in the macaque brain (P100 at ∼80 ms; P200 at ∼200 ms) and a negativity (N100 at ∼110 ms), see Fig. 2. We also observe that even the response to the last element showed clear N100, P100, and P200 ERP components (Fig. 2C).

**Fig. 3:**
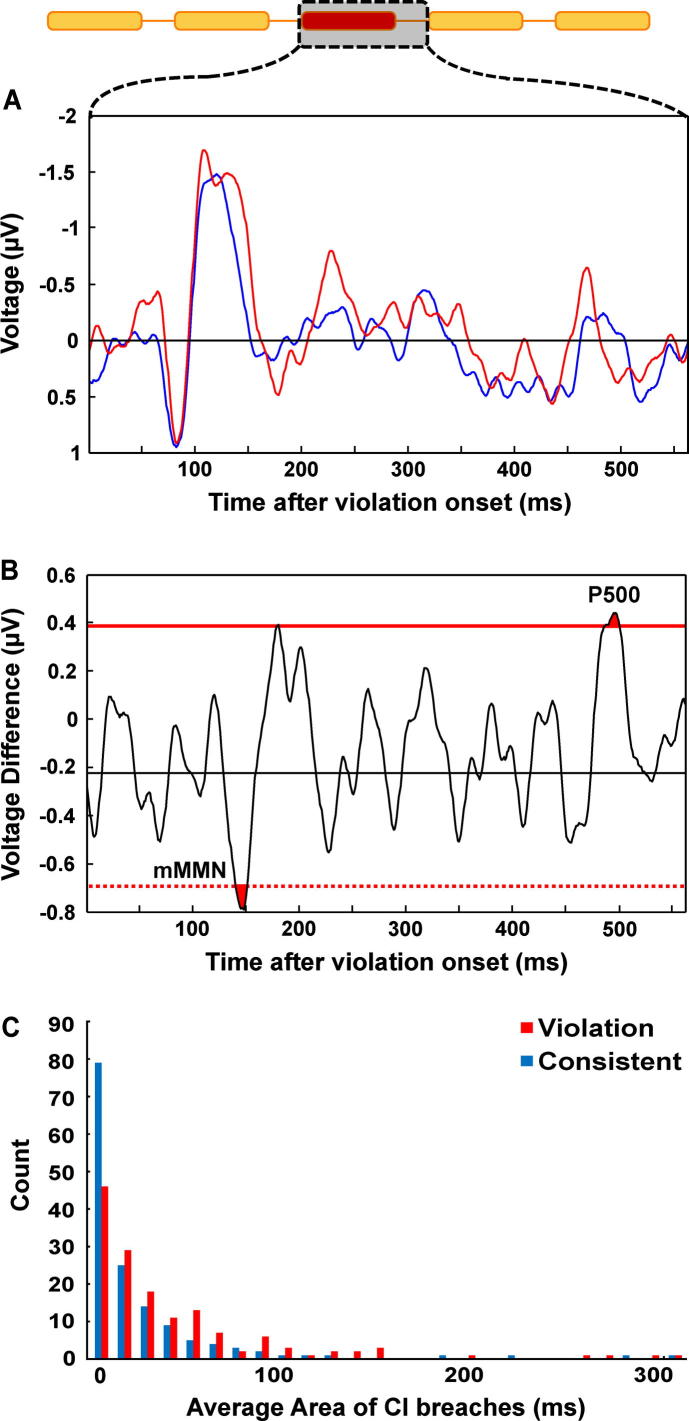
Artificial grammar sequence processing effects in the frontal electrodes following violation sound onset. (A, B) Grand average ERPs across the frontal electrodes (FP1, FP2, F3, F4), aligned to the onset of a violation within the 5 element sequence (see Fig. 1 and Suppl. Figs. S1–3). ERPs shown are for the first illegal sound element and its matching consistent sequence element. (A) Grand average ERP for consistent responses (blue line) and violation responses (red line). (B) Difference plot (violation minus consistent, see A). Red lines correspond to the upper (solid) and lower (dashed) bounds of the 95% confidence interval (2.5% and 97.5% respectively), defined by the variability in the difference waveform during a 613 ms baseline period including the 200 ms prior to the start of the first sound in the sequence through the end of the first element in the sequences, which was always element ‘A’ (see Section 2 for further details). Black horizontal line shows the mean of the baseline period difference waveform, reflecting some variability in the difference waveform in response to the violation minus consistent sequence elements. Areas that breach the confidence interval are filled in red. (C) Histogram showing the distributions of average area of CI breaches (2 comparison pairs per session, for all the sessions with each macaque; *n* = 148 data points).

Next we evaluated effects related to the AG sequencing condition (consistent or violation) by analysing ERP differences to the corresponding consistent and violation comparison sequence pairs. Namely, the first violating sound present in the violation sequence had an acoustically identical match in its consistent sequence pair (Fig. 1C). Fig. 3A shows the ERP components to the violation and consistent sequences in response to the violating sound in the violation sequences, for the frontal electrodes where we expected certain ERP components to be more prominent (Suppl. Fig. S4 shows the grand average ERPs for all electrodes). To identify statistically significant effects we first created a difference waveform (violation minus consistent; Fig. 3B). We then determined the lower and upper bounds of the 95% confidence interval (CI) from the difference waveform variability during a baseline period; the baseline period was defined as the silent period before the start of the sound sequences and the first element in the sequences, which was always element ‘A’ (Suppl. Figs. 2 and 3). Projecting the CI over the period during the violation element and its corresponding consistent sound pair (Fig. 3) was used to identify significant waveform differences that deviate in preference for either the violation or consistent condition. This analysis identified both positive and negative differences between the violation and matched consistent sequence elements. In the four frontal electrodes (FP1, FP2, F3, F4), the violation condition elicited a stronger early negativity peaking at 148 ms (see red trace in Fig. 3A and the first breach in the difference waveform in Fig. 3B). This early ERP component resembles a macaque homologue of the human MMN (which we will refer to as the mMMN; Fig. 3B). In these frontal set of electrodes, the violation condition also elicited a later more positive ERP peaking at 497 ms (Fig. 3A and B). The grand average ERP across all of the electrodes also show a strong mMMN (Suppl. Fig. S4), although in the results with all electrodes combined the P500 is weaker and we identify another early component (P200). Interestingly, these effects were specific to the first violation sound because no obvious differences between the violation and consistent sequences were evident for the subsequent sound after the violation (Suppl. Fig. S5; note here that all three ERP components, the mMMN, P200 and P500 are evident in this analysis only including the sequences which had at least two sounds after the violation that could be comparably analysed between the violation and consistent sequences).

We evaluated whether there was a session-by-session ERP response preference either for the violation or consistent conditions. Here, we analysed the session-by-session breaches across the confidence interval (CI) over the period including the violation sound and its matching consistent sound element (563 ms). We measured the number and average area (using trapezoid method) of the CI breaches in favour for either the violation or consistent condition. We observed that the distribution of average area breaches across the CI was shifted towards significantly higher areas for the violation, relative to the consistent sequences (Fig. 3C; Wilcoxon signed-rank test, *p *= 0.013; mean CI breach area (standard error of the mean, SEM), for violation: 12.81, (±1.96); for consistent: 8.73 (±1.17)). The violation sequences also elicited a significantly greater number of breaches above the confidence intervals than the consistent sequences (Wilcoxon signed-rank test, *p *< 0.001; mean number of breaches, for violation: 2.85 (±0.18); consistent: 2.07 (±0.16)).

We used a Repeated Measures Analysis of Variance (RM-ANOVA) to investigate whether the identified ERP components (mMMN, P200 and P500) were left or right hemisphere lateralized, or distributed more anterior or posterior on the head. First we identified the time windows of interest for each of these components based on our results with the CI analyses (Fig. 3B, Suppl. Fig. S4B). Next a 40 ms response window was centred on the position of each of these identified ERP components (MMN: 128–168 ms; P200: 161–201 ms; P500: 480–520 ms). Within these windows, the maximum value of the EEG response to both the consistent and violation sequences was measured. These values were submitted to the RM-ANOVA containing a within subjects factor of Condition (consistent or violation). Also between subjects factors of ‘monkey’ and two additional between subjects factors identifying the position of the electrodes were modelled (‘Antero-posterior axis’ and ‘Hemisphere’ (left/right)).

With the RM-ANOVA results, first we confirmed that there were significant main effects for Condition for all of the noted ERP components (mMMN: *F*_(1,1176)_ = 9.607, *p *< 0.001; P200: *F*_(1,1176)_ = 5.392, *p *< 0.001; P500: *F*_(1,1176)_ = 5.058, *p* = 0.025). The amplitude of the EEG responses differed by macaque for many of the factors, as might be expected (main effect of Macaque; mMMN: *F*_(1,1176)_ = 9.443, *p* = 0.002; P200: *F*_(1,1176)_ = 75.008, *p *< 0.001; P500: *F*_(1,1176)_ = 144.503, *p *< 0.001). Notably however the P500 and P200 component did not have a significant Condition by Macaque interaction, suggesting that the macaques did not differ in the effects for these components (P200: *F*_(1,1176)_ = 1.731, *p* = 0.188; P500: *F*_(1,1176)_ = 0.872, *p* = 0.351). The mMMN did show a Condition by Macaque interaction (mMMN *F*_(1,1176)_ = 9.607, *p* = 0.02). However, the polarity of all ERP components (mMMN, P200 and P500) was consistent across the macaques (Suppl. Fig. S8). Regarding topographical distribution, only the mMMN component was significantly different between left- and right-hemisphere channels: the mMMN was stronger in the left (FP1, F3, C3, P3) than right electrodes (FP2, F4, C4, P4), (*F*_(1,1176)_ = 4.199, *p* = 0.041). Regarding anterior or posterior head distribution, the RM-ANOVA showed that the mMMN, P200 and P500 components were all significantly stronger in the more anterior electrodes (electrodes FP1, FP2, F3, F4; mMMN *F*_(1,1176)_ = 48.893, *p *< 0.001; P200, *F*_(1,1176)_ = 6.649, *p* = 0.01; P500, *F*_(1,1176)_ = 5.033, *p* = 0.025).

To match the violation sequences with the same elements in consistent sequences involved shifting the alignment of some of the sequences. We confirmed that the reported effects were evident in the grand average ERP response including sequences that were not shifted and those that were balanced in the direction of shifting (Suppl. Fig. S6). Also, since we required that the sounds after a violation were acoustically matched, the experimental design necessitated that the sounds preceding the violation were different between consistent and violation sequences (Fig. 1C). Thus we asked whether the magnitude of the response difference to these sounds prior to the violation was associated with the size of the mMMN and P500 ERP components seen in response to the violation sound. This could identify an interesting contextual effect whereby the strength of the acoustically-related EEG response to the consistent vs. violation sequence element prior to the violation was associated with the strength of the effect to the violation sound and its acoustically matched consistent sequence sound (illustrated in Suppl. Fig. S7C). However, there was no significant association with the magnitude of the response difference to the acoustically different sounds prior to the violation and the magnitude of the mMMN and P500 effects to the violation sound (Suppl. Fig. S7).

## 4 Discussion

This macaque EEG study provides evidence that certain ERP components are modulated by violations to a moderately complex, finite-state AG, which macaques appear able to implicitly learn (Wilson et al., 2013). We observed that violations to adjacent relationships in the AG modulated positivities and negativities (from ∼150–500 ms), with the strongest modulations occurring for the macaque mMMN, P200 and a later frontal positivity (P500). We next separately discuss each of these observed ERP components in relation to the literature, including ERPs reported in human EEG studies of AG learning.

Our experimental design ensured that effects related to a violation sound were analysed in relation to an acoustically identical sound in a matched comparison consistent sequence. Thus the results cannot easily be attributed to acoustical differences and instead reflect the sequencing condition in which the nonsense word elements occurred (i.e., whether the order of the preceding elements in the sequence lead to the analysed element being consistent with or in violation of the AG). We observed that for a violation element, certain ERP components were modulated to a greater extent than for their acoustically matched elements in the consistent sequence. The effects were restricted to the violating element, as none of the effects persisted for the next sound following the violation element. Moreover, the reported effects are evident in sequences with no shifting and balanced shifting between consistent and violation sequences (Suppl. Fig. S6). Furthermore, the reported effects do not appear to be associated with the acoustically-related EEG response to the sound prior to the violation (Supp. Fig. S7).

We had hypothesised that AG violations would modulate a number of components, such as the monkey homologs of the MMN (Bekinschtein et al., 2009; Fishman & Steinschneider, 2012; Gil-da-Costa et al., 2013; Javitt et al., 1992; Naatanen & Alho, 1995 ; Ueno et al., 2008; Uhrig et al., 2014; Ulanovsky et al., 2003), human Early Left Anterior Negativity (ELAN: Friederici, 2004; Friederici et al., 2002), P200 (Garcia-Larrea et al., 1992; Novak et al., 1992) and P3a (Arthur & Starr, 1984; Baldwin & Kutas, 1997; Gil-da-Costa et al., 2013; Molholm et al., 2005; Mueller et al., 2012; Paller et al., 1988; Pineda et al., 1988; Zevin et al., 2010). Two of these predictions were met. We observed mMMN and P200 ERP components. The mMMN is of interest because human EEG studies using AGs with similar levels of complexity to the AG used here (e.g., those with adjacent relationships) can elicit an MMN (Baldwin & Kutas, 1997; Mueller et al., 2012). However, such AGs also often elicit an ELAN (Friederici, 2004; Friederici et al., 2002), for which we found no strong evidence of a macaque homolog using our significance criteria, although care is needed when interpreting topographical distributions from our limited set of electrodes. Interestingly, we observed a prominent late P500 component, which was not predicted because such a late positivity in human AG learning studies is usually associated with more complicated (e.g., non-adjacent) AG relationships (Friederici, 2004; Friederici et al., 2002).

Some of the earlier macaque ERP components show considerable similarities to those that have been reported in the EEG literature, in relation to ERP components modulated by violations of expectancy. Prior human and nonhuman animal work studying neuronal responses or ERPs, associated with oddball sounds or change detection, have also noted effects on early components. For example, the nonhuman animal homolog of the MMN response is an extended negativity occurring at ∼150 ms after stimulus onset, which is elicited by an acoustically deviant sound (Bekinschtein et al., 2009; Fishman & Steinschneider, 2012; Gil-da-Costa et al., 2013; Javitt et al., 1992; Naatanen & Alho, 1995 ; Ueno et al., 2008; Ulanovsky et al., 2003). Human EEG studies of AG learning have also reported effects on the MMN or P300 when the violation sequences are presented infrequently (Baldwin & Kutas, 1997; Mueller et al., 2012). Thus, our observed enhancement of the macaque mMMN and P200 by the AG violation condition could relate to violations of expectancy. However, our study ensured balanced presentation of violation and consistent sequences, which is also the case for many other AG learning studies (Friederici, 2004; Friederici et al., 2002). Thus, the violation of expectancy in this experiment was established by the period of exposure (lasting for 30 min) prior to testing.

The P500 component that we observed was unexpected but appears to be a robust effect. The later positivity to AG violations in humans (P600) might be near enough in time to be a homolog of the component that we see in the macaque P500. However, the P600 in humans is thought to be elicited by more complex AGs including those that have hierarchical relationships of the forms that are only present in human language (Friederici, 2002, 2005) and for which there is currently no clear evidence that any nonhuman animal can learn (Berwick, Okanoya, Beckers, & Bolhuis, 2011). Therefore, our macaque P500 is unlikely to be a direct homolog of the P600 reported in humans for complex AG or syntax-related processes. It also seems unlikely that the observed macaque P500 is a later macaque homolog of the P3a since the macaque P3a in response to oddball sounds does not seem to persist through to 500 ms after sound onset (Gil-da-Costa et al., 2013). Furthermore, during the time period where we might expect a macaque P3a component (200–350 ms), we see, if anything, a stronger negativity for the violation sequences (Fig. 3). This observation is inconsistent with our observed P500 being a remnant of a prior sub-threshold P3a effect. Thus, the correspondences in the polarity and general time of occurrence of the macaque P500 to the reported human P600 might reflect evolutionarily conserved processes involved in evaluating sequences for consistency with previously learned sequencing relationships. The differences in the functional role for the macaque P500 and the human P600 may reflect the differentiation that has occurred in humans to support language-specific processes.

In conclusion, we identified a number of expected effects on early macaque ERP positivities and negativities associated with AG learning, such as prominent effects on the macaque mMMN and P200. We did not identify a corresponding homolog of the human ELAN response to adjacent AG violations. However, we note a prominent later frontal positivity (P500), which although unexpected is similar in polarity and relative time of occurrence, but likely differs in its functional role, to the P600 that has been reported in human EEG studies of more complex forms of AG learning. This first macaque EEG study of AG learning raises the possibility that certain processes associated with auditory sequence analysis are evolutionarily conserved as reflected in the ERP responses that were measured here. Some, like the macaque P500, might have further functionally differentiated in humans. The conserved aspects of the ERP components can now be studied at the neuronal level in macaques, as a primate model system, and related to humans using comparative EEG and fMRI.

## Author contributions

A.A. and C.I.P. designed research; A.A. and A.M. performed research; A.A. analysed data; B.W., K.A., Y.K., and C.I.P. provided materials and intellectual contributions; A.A. and C.I.P. wrote the paper with input from the co-authors.
