# Supplementary material for: EEG potentials associated with artificial grammar learning in the primate brain
Source: Brain Lang. 2015 Sep;148:74–80. doi: 10.1016/j.bandl.2014.11.006 (PMC4557543; doi:10.1016/j.bandl.2014.11.006)
Supplement: Supplementary data 1 [file mmc1.docx]

**Supplementary Materials** for Attaheri et al*.*, ‘EEG potentials associated with artificial grammar learning in the primate brain’

**Contents:**

**I. Time course of experimental trial** (Suppl. Fig. S1). 2

**II. Exposure and testing sequences** (Suppl. Fig. S2) 3

**III. Comparison pairs of consistent and violation AG sequences** (Suppl. Fig. S3). 4

**IV. Grand average ERPs across all electrodes** (Suppl. Fig. S4) 5

**V. Effects occur for the violation sound but do not generalize to the subsequent sound** (Suppl. Fig. S5). 6

**VI.** **Effects for sequences balanced in the direction of shifting** (Suppl. Fig. S6). 8

**VII. Violation-related effects do not seem to depend on the response to the sound prior to the violation** (Suppl. Fig. S7). 10

**VIII. ERPs by macaque** (Suppl. Fig. S8). 12

**IX. Peak-voltage responses for each macaque and condition.** (Table S1). 13

**I. Time course of experimental trial (Suppl. Fig. 1)**


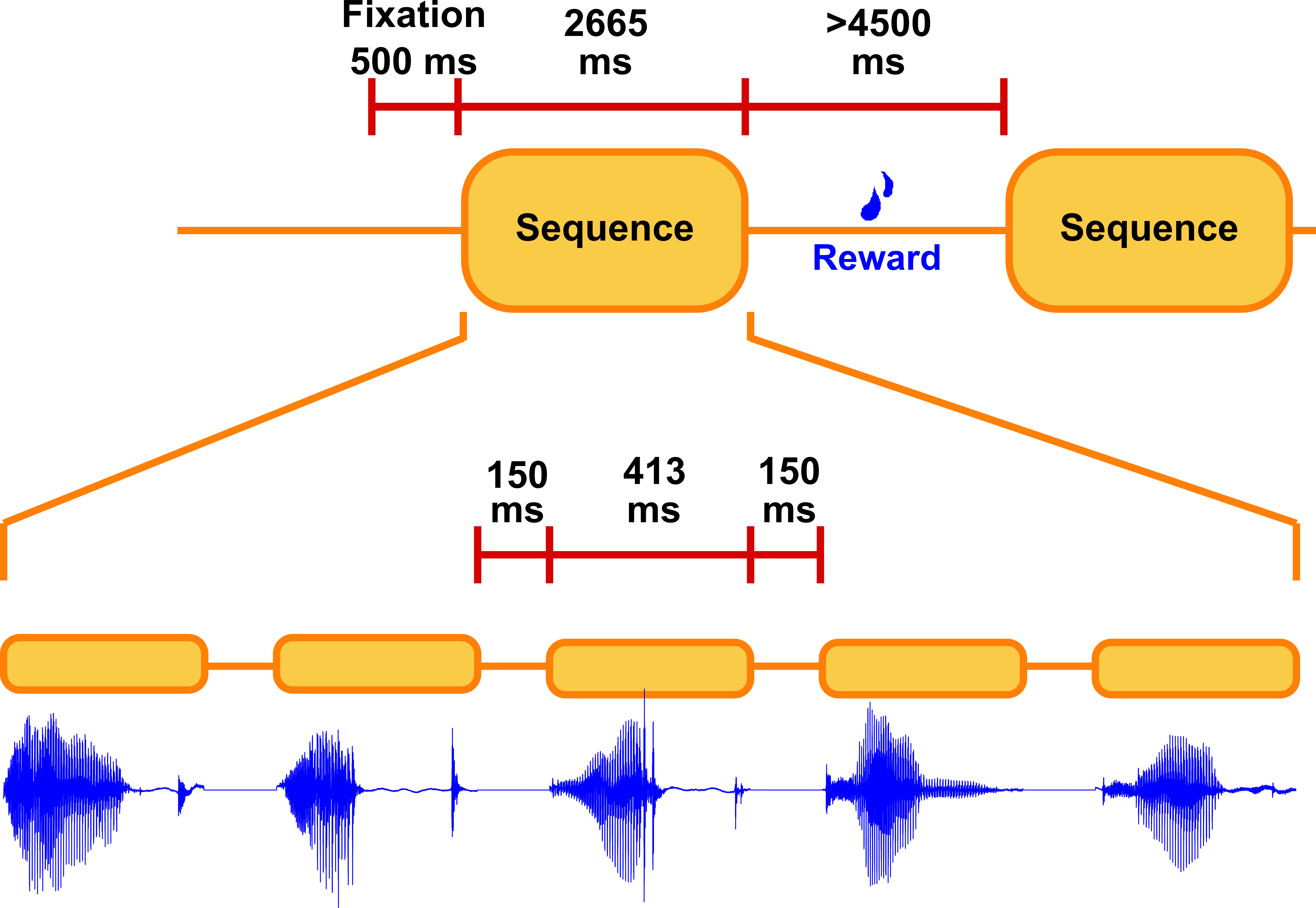


**Supplementary Figure S1. Time course of experimental trial.** Schematic of an experimental trial. Sequences consisted of five acoustic elements. Each element was a 413ms, consonant vowel consonant, nonsense word separated by 150ms inter stimulus interval (ISI). A sequence was initiated by the macaque fixating a visual spot on the monitor screen for 500ms. Each sequence was separated by minimally a 4500ms inter trial interval (ITI). The ITI could vary because the start of the next trial depended on when the macaque engaged the fixation spot.

**II. Exposure and testing sequences (Suppl. Fig. S2)**

Eight exposure sequences were presented in a random order to the animals for 30 minutes prior to testing (Suppl. Fig. S2). During the subsequent testing phase, four ‘consistent’ and four ‘violation’ sequences were randomly selected without replacement and individually presented to the animals during the testing trial while the EEG signal was recorded (Suppl. Fig. S1). Some violation sequences (C3-C4) could have multiple violations, but for this study, analysis was only conducted on effects related to the first sound that violates the AG (which we will refer to as the ‘violating sound’).

**
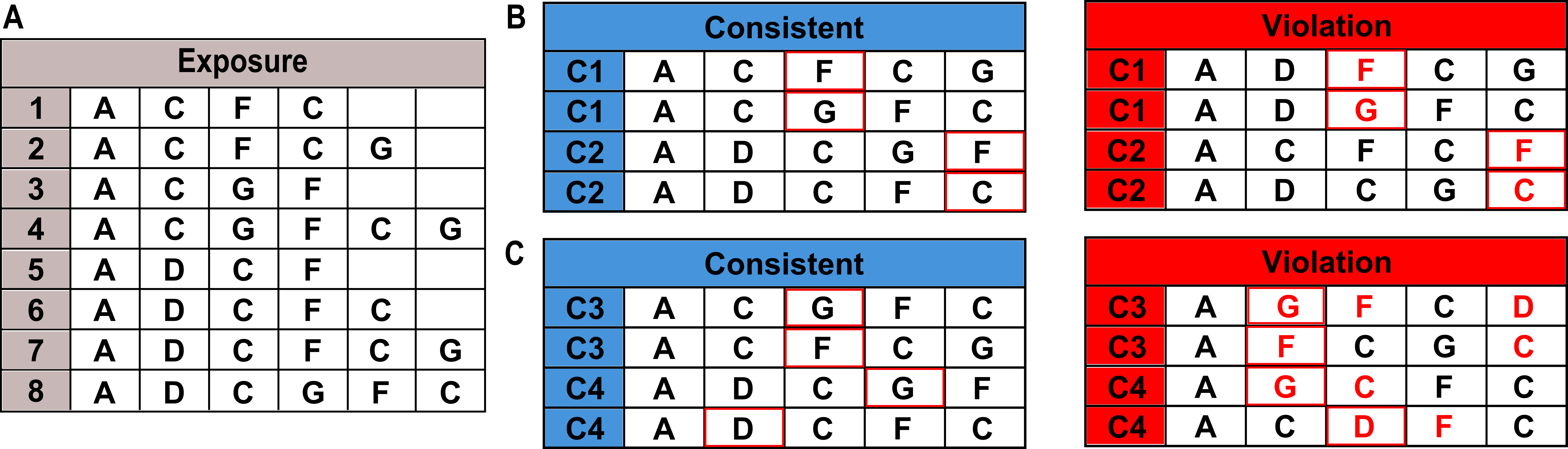
**

**Supplementary Figure S2. Exposure and testing sequences**. (A) 8 sequences used in the exposure. (B-C) Testing sequences used. (B) All sequences used in the Block A data acquisition experiment, 4 consistent and 4 violation sequences (see Methods for further details). Red letters denote the first violation element (first element after an illegal transition). The red boxes show the comparison sound element pairs used for comparing effects between the consistent and violation sequence pairs. (C) The 4 consistent and 4 violation sequences from the Block B data acquisition experiment.

**III. Comparison pairs of consistent and violation AG sequences. (Suppl. Fig. S3)**

Illegal transitions in the violation sequences occurred at different time points in the sequences. To ensure identical elements were being compared, consistent and violation sequences were analysed as aligned comparison pairs during which the same sound could be compared across the two conditions. The condition (violation vs. consistent) was identified by whether the transition from the preceding sound to the sound being analyzed (identified by the 563ms red line) was illegal (not allowed by the AG) or legal (allowed by the AG), see Suppl. Fig S3.


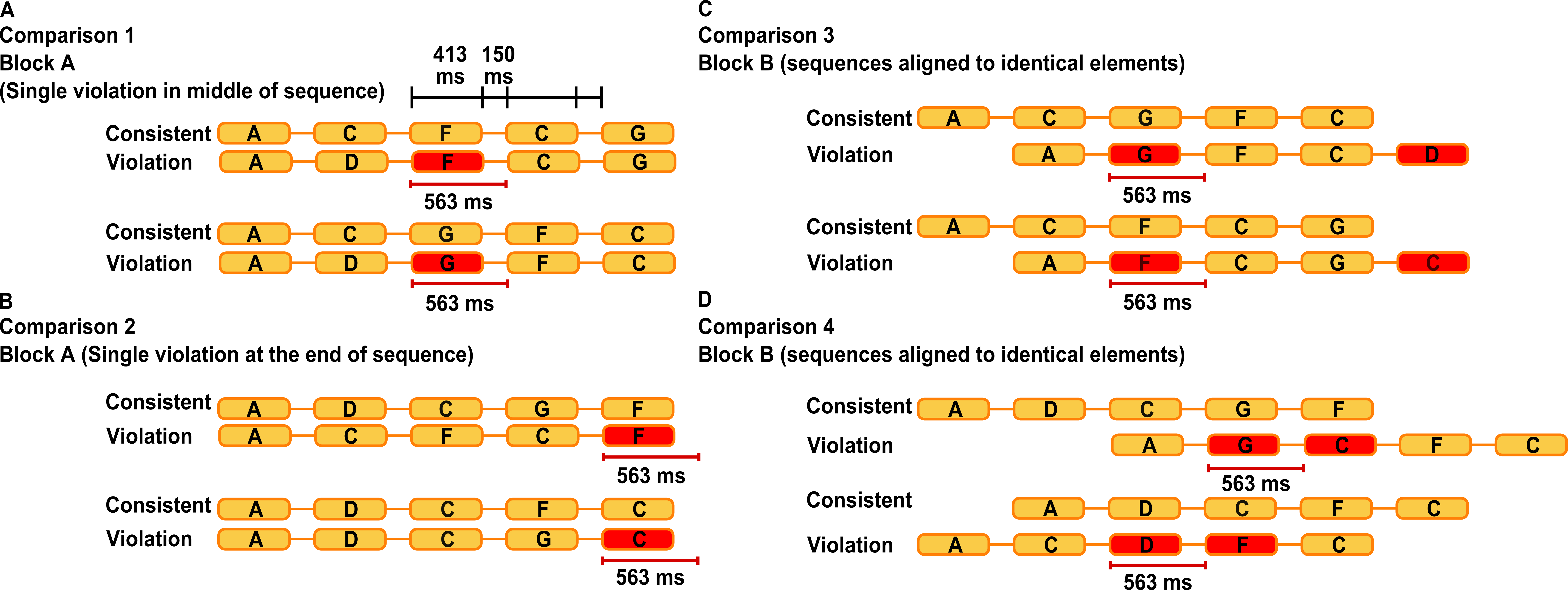


**Supplementary Figure S3. Sequence comparison pairs.** Schematic of the consistent and violation sequences used in the analyses. Letters A, C, D, F, G represent the auditory elements of the AG shown in Suppl. Fig. S2 (also see manuscript Fig. 1). Red boxes highlight the first violation element. All violation sequences are aligned and paired to their matching consistent sequence pairs. (A, B) Sequences taken from data acquisition Block A (see Methods), illustrating the 4 consistent and 4 violation sequences with a single violation in the middle or end of the sequence respectively. (C, D) Sequences taken from data acquisition Block B, illustrating the 4 consistent and 4 violation sequences. All sequences are aligned so that the first violation element in the violation sequence is compared to an acoustically identical element in the matching consistent sequence pair.

**IV. Grand average ERPs across all electrodes (Suppl. Fig. S4)**

Fig. 3 in the main manuscript presents the data from the frontal electrodes only, since we had hypotheses focusing on the frontal electrodes (FP1, FP2, F3, F4). The grand average ERP from all electrodes was also used to identify effects and to define the time points of maximal breach from the confidence intervals (Supp. Fig. S4).


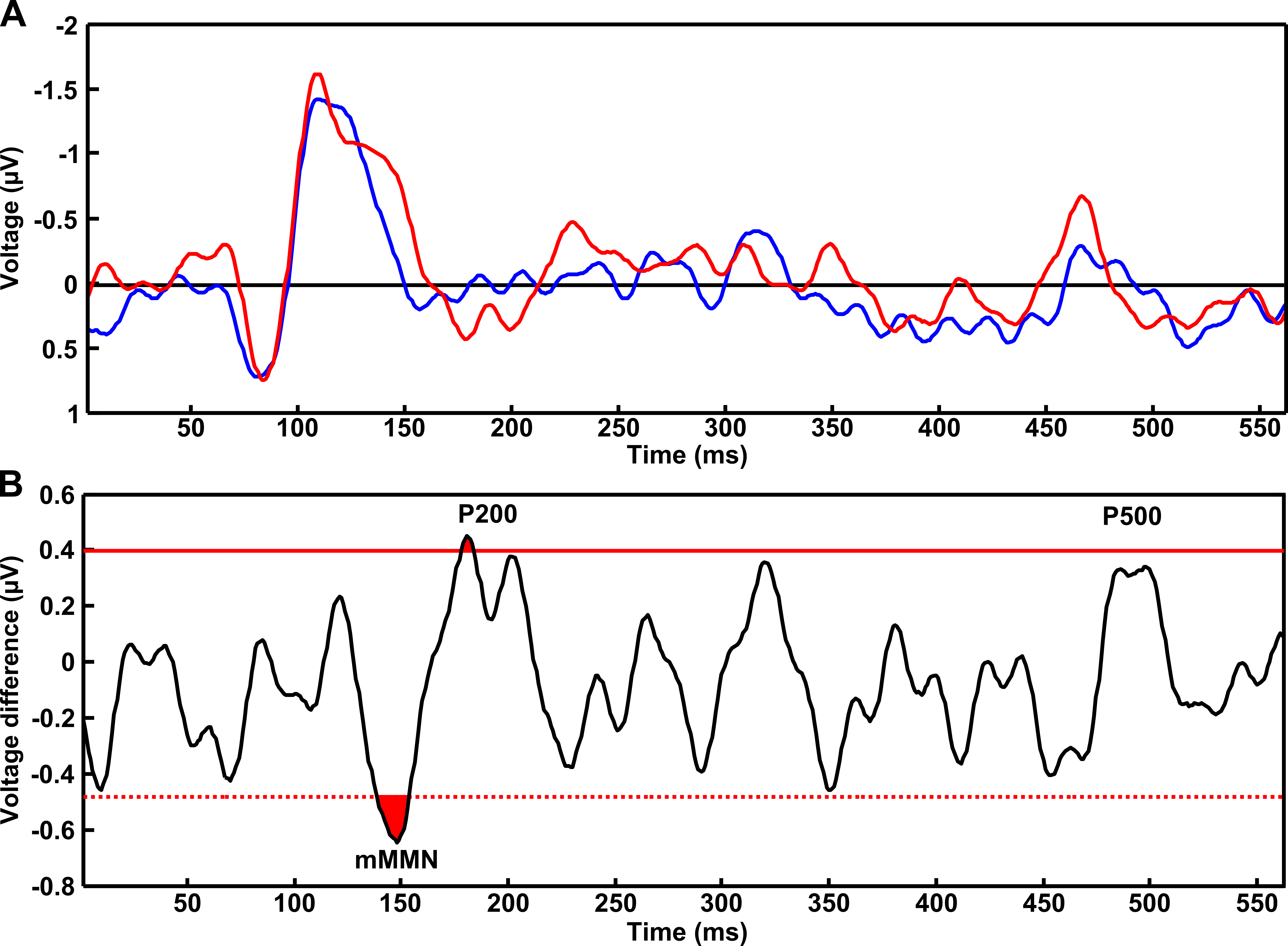


**Supplementary Figure S4. Grand average ERP across all eight electrodes**. (A) Grand average of consistent (blue) and violation (red) sequences from all electrodes (FP1, FP2, F3, F4, P3, P4, C3, C4) from both macaques. Data was aligned to the onset of the violation sound in the violation sequences and the matching consistent sequence sound. (B) Difference plot (violation minus consistent) of the grand average in (A). Red solid line corresponds to the upper bound of the 95% confidence interval and the red dotted line corresponds to the lower bound of the 95% confidence interval (see Methods). Periods that breached the CI are highlighted in red and periods of interest (also in relation to Fig. 3 in the manuscript) are labelled, mMMN, P200, and P500.

**V. Effects occur for the violation sound but do not generalize to the subsequent sound (Suppl. Fig. S5)**

To see if the ERP components were elicited solely for the first illegal element/sound, or also occur in the later sounds, we evaluated effects to the violation sound and the subsequent sound. To ensure no acoustical differences confounded the result, analysis was only performed on sequences where all the elements after violation were identical in the ‘consistent’ and ‘violation’ sequences (i.e. comparison pairs one and three, see Suppl. Fig. S2A and S2C). Results showed that during the first violation element the mMMN, P200 and P500, ERP components were significantly different between the consistent and violation condition, as shown by the CI breaches in favor of the violation condition (Suppl. Fig.S5A-B). However no significant difference in the ERP components remained for the next sound (Suppl. Fig. S5C-D).


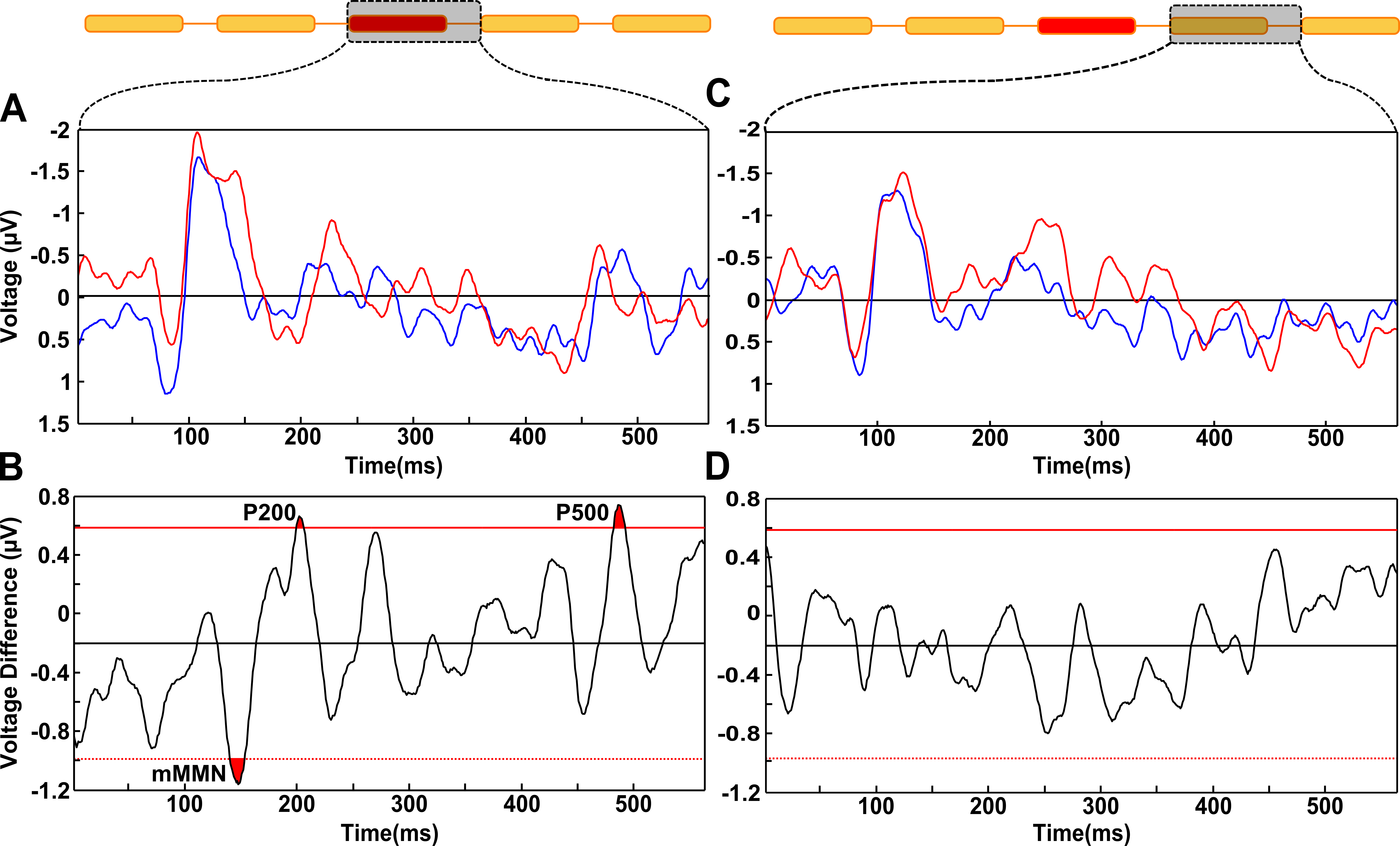
**Supplementary Figure S5. Comparison of ERP differences to violation vs. consistent sequences in the first violation sound and the sound subsequent to it.** (A) Grand average ERP for comparison pairs 1 and 3 from the frontal electrodes (FP1, FP2, F3, F4), aligned to the onset of the first violation element. Consistent (blue) and violation (red) response waveforms are shown. (B) Difference plot (violation minus consistent) aligned to the onset of the violation element. (C) Grand average ERP, for comparison pairs 1 and 3 from the frontal electrodes (FP1, FP2, F3, F4), aligned to the subsequent element after violation element, see Suppl. Fig 3. (D) Difference plot (violation minus consistent) aligned to the subsequent element after the violation element. (B,D) The solid red line corresponds to the upper bound of the 95% confidence interval and the dotted red line corresponds to the lower bound (see Methods). Black centre line shows the mean of the baseline period difference waveform. Areas that breach the confidence interval are filled in red.

**VI. Effects for sequences balanced in the direction of shifting (Suppl. Fig. S6)**

Shifting the sequences to allow direct comparisons of identical acoustical elements could be expected to affect the results because of greater/weaker adaptation in some shifted sequences being compared. This would predict that even earlier components (such as the N1/P1) would show obvious differences between the conditions. However, we do not see any evidence for the earliest ERP components (N1/P1) being affected in any of our results (manuscript Fig. 3; Suppl. Fig. S4). Nonetheless, here we evaluate the effects on sequences balanced in the direction of shifting. This analysis includes sequences where there was no shifting and ones where the shifting is matched in direction between the consistent and violation sequences; see panel C in Suppl. Fig. S6. The results of this analysis are shown in Suppl. Fig. S6A-B. Although we lose statistical power for this analysis at least the mMMN and P500 effects are clearly visible just below the 95% bounds and are well within the 90% bounds (statistical trend). This suggests that imbalanced shifting cannot easily account for the main effects reported in the paper.


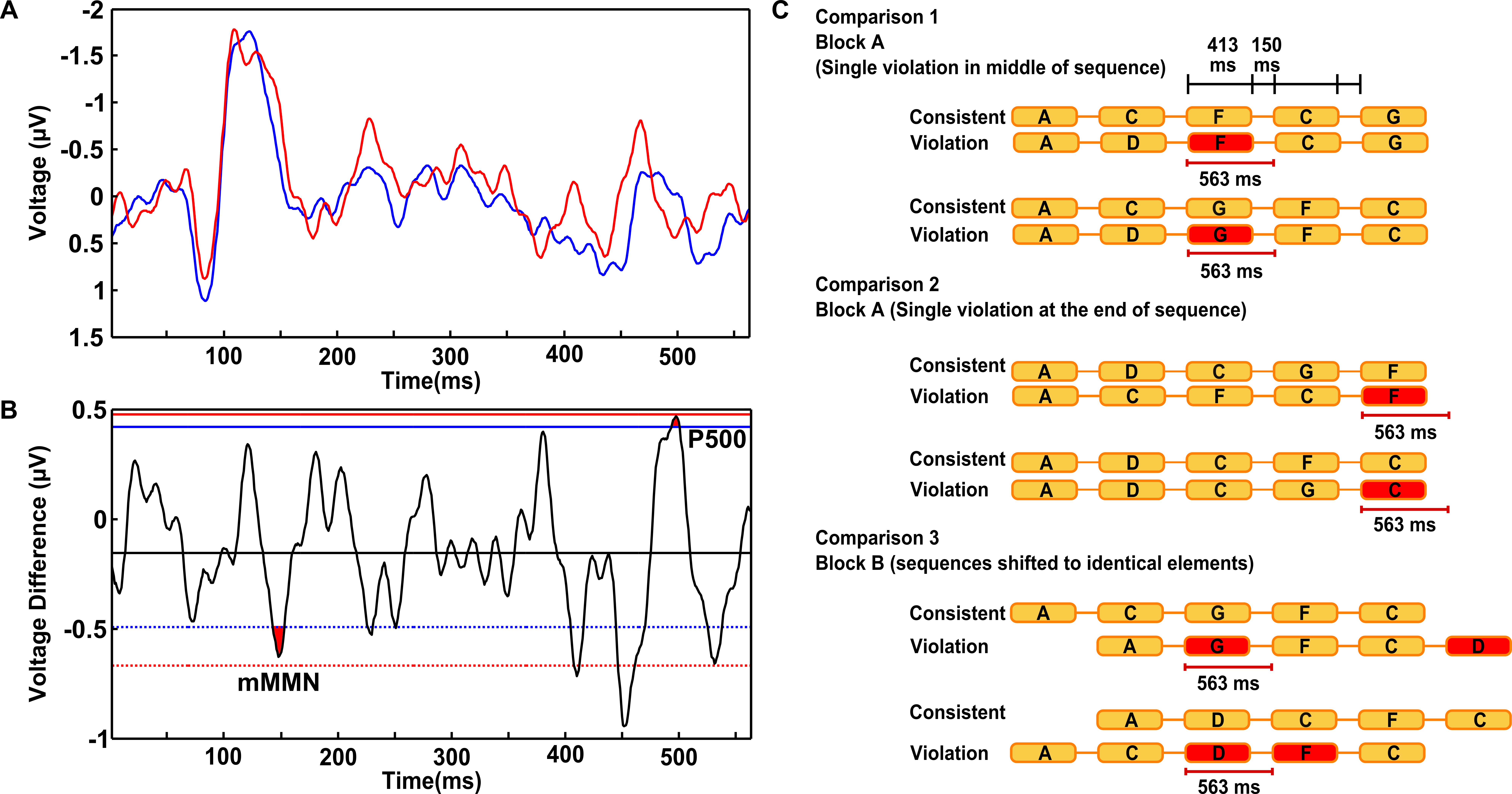


**Supplementary Figure S6. Balancing for direction of shifting.** (A) Grand average ERP for the frontal four electrodes (FP1, FP2, F3, F4) across the two macaques, aligned to the onset of the first violation element in the sequence. Grand average ERP for the consistent (blue) and violation (red) sequences during the 563ms analysis window shown in (C). (B) Difference plot (violation minus consistent) of the grand average in (A). Red lines indicate the upper and lower bounds of the 95% CI and blue lines show the upper and lower bounds of the more liberal 90% CI. The mMMN and P500 effects labelled and highlighted in red. (C) Shows the comparison pair sequences that were used for these analyses (A).

**VII. Violation-related effects do not seem to depend on the response to the sound prior to the violation (Suppl. Fig. S7)**

An interesting question is whether the reported ERP components are influenced by the way that the brain responds to the sound prior to the violations (which are acoustically different between the violation and consistent sequences). This could identify contextual effects to the reported ERP components, associated with the strength of the acoustically-related EEG response difference to the sound preceding the violating sound. Here we analysed the association between the difference in the EEG response to the correct vs. violation sounds prior to the violation and the strength of the reported mMMN and P500 effects. This is illustrated in Suppl. Fig. S7C. The correlations between the mMMN and P500 effects after violation do not show a significant association with the response difference (absolute mean of the difference waveform) to the sound prior to the violation in the violation vs. consistent sequences (mMMN: *r* = -0.045, *p* = 0.705; P500: *r* = -0.125, *p* = 0.289, N=74, Pearson correlation). These findings suggest that the reported contextual effects are not simply driven by the ERP response to the acoustically different sounds prior to the violation.


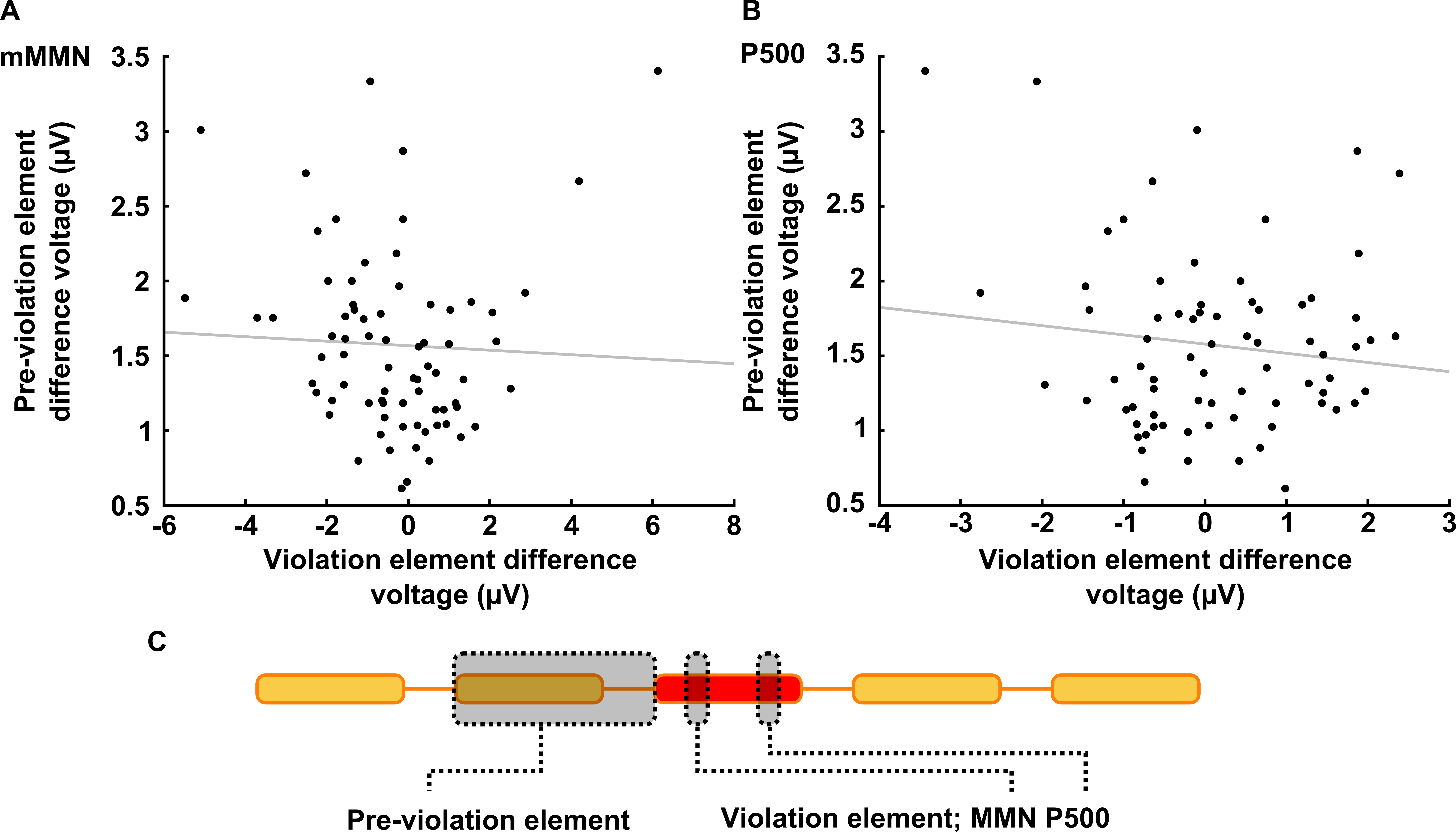


**Supplementary Figure S7. Violation-related effects do not seem to depend on the response to the sound prior to the violation.** (A) Correlation between the mean absolute difference (violation minus consistent) in the EEG response to the sound prior to the violation (illustrated in C) and the magnitude of the mMMN in response (violation minus consistent) to the violation sound. This analysis is conducted across sessions and macaques (N=74). (B) Analysis evaluating this correlation for the P500. (C) Schematic showing the time windows from which the data was extracted for these analyses.

**VIII. ERPs by macaque** (Suppl. Fig. S8)


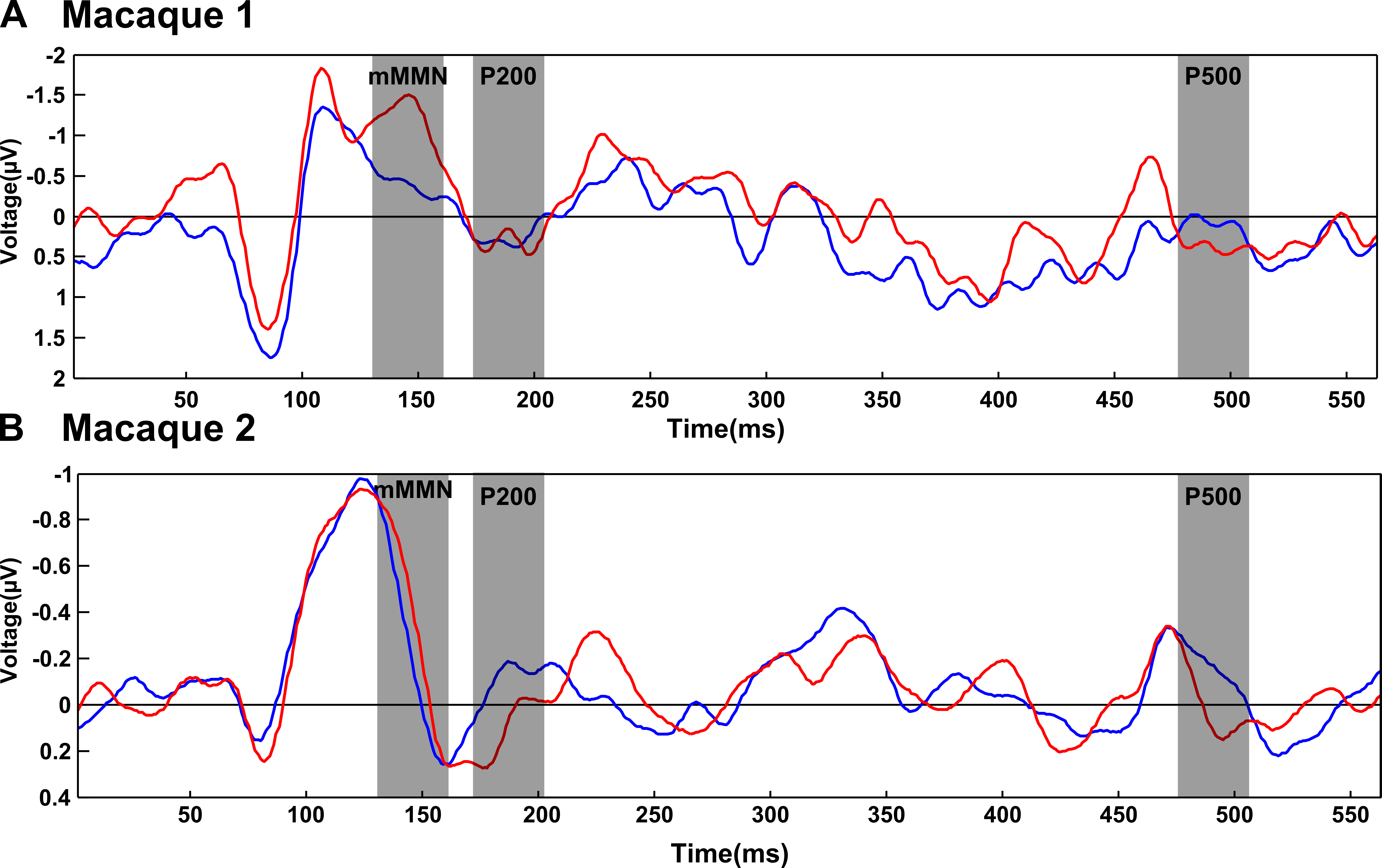


**Supplementary Figure S8. Individual macaque ERPs.** (A) Grand average ERP for macaque 1 (M1) showing consistent (blue) and violation (red) sequences from the frontal electrodes (FP1, FP2, F3, F4). (B) Grand average ERP for macaque 2 (M2) showing consistent (blue) and violation (red) sequences from the frontal electrodes. The grey boxes illustrate the time frames of interest in both macaques, corresponding to the mMMN, P200, P500.

**IX. Separate macaque ERP voltages**

**Table S1. Average peak ERP voltage responses across sessions, shown separately by macaque and condition (consistent or violation).**

For this analysis, components of interest were obtained from the grand average difference plots (Fig. 3 and Suppl. Fig.S4) by placing a 40ms response window around each peak difference exceeding the CI (mMMN: 128-168ms; P200: 161-201ms; P500: 480-520ms). These time windows were next evaluated session by session to extract the maximum voltage in response to the consistent and violation sequences. The resulting values reported in the table are averaged across the sessions. Note that the resulting voltage values need not correspond to the average magnitude values reported in the manuscript. Both methods show that the violation elicits a more negative voltage in both monkeys for the mMMN component and that the violation elicits more positive potentials in both monkeys for the P200 and P500. Note that the polarity of the different components is consistent across the two animals.

|  | mMMN | | P2 | | P5 | |
| --- | --- | --- | --- | --- | --- | --- |
|  | **Consistent** | **Violation** | **Consistent** | **Violation** | **Consistent** | **Violation** |
| **M1** | -2.341 | -2.963 | 2.194 | 2.460 | 2.511 | 2.602 |
| **M2** | -2.301 | -2.411 | 1.371 | 1.828 | 1.402 | 1.621 |
